# Regulation of viable/inactivated/lysed probiotic *Lactobacillus plantarum* H6 on intestinal microbiota and metabolites in hypercholesterolemic mice

**DOI:** 10.1038/s41538-022-00167-x

**Published:** 2022-10-31

**Authors:** Yue Li, Mengling Chen, Yuxuan Ma, Yue Yang, Ying Cheng, Huijing Ma, Dayong Ren, Ping Chen

**Affiliations:** College of Food Science and Engineering, Jilin Agricultural University, 130118 Changchun, China

**Keywords:** Applied microbiology, Bacterial secretion

## Abstract

Evidence suggests that probiotic interventions reduce non-communicable diseases (NCDs) risk. However, its therapeutic effect and mechanism are still unclear. To evaluate the hypocholesterolemic effect of *Lactobacillus plantarum* H6 (*L.p* H6), a new commercial patent strain capable of preventing hypercholesterolemia, and its mechanism in depth, three states of the strain were prepared, namely, viable (vH6), heat-inactivated (iH6), and ultrasonically-lysed (uH6) bacteria cells. The results showed that v/i/uH6 cells could lower serum and liver blood lipid levels, alleviate liver damage and improve glucose tolerance test (GTT) and insulin tolerance test (ITT) indexes. v/i/uH6 cells improved the gut microbial composition and significantly reduced the *Firmicutes* to *Bacteroidetes* ratio (F/B ratio) in feces. In particular, *Muribaculaceae* may be a potential biomarker for effective cholesterol reduction. Also, the recovery of these biochemical indices and gut microbiome was found following fecal microbiota transplantation (FMT) using stool from vH6 treated mice. The v/i/uH6 cells increased the intestinal flora metabolism of vitamins-cofactors, as well as amino acids, while decreasing the relative content of primary bile acids. The Pearson correlation analysis showed that *norank_f__Muribaculaceae* and *Lactobacillus* had a negative correlation with blood lipid levels. Overall, v/i/uH6 cells were effective in improving hypercholesterolemia in mice, and this effect was attributed partly to the regulation of intestinal microbiota and metabolites related to lipid metabolism. Our findings provided a theoretical basis for the industrial development of probiotics and postbiotics and the treatment of cholesterol diseases.

## Introduction

Cholesterol level in the human body plays a vital role in normal cell and function, but excessive levels can lead to lipid metabolism problems and increase the non-communicable diseases (NCDs) risk, such as cardiovascular disease (CVD), nonalcoholic steatohepatitis (NASH), obesity, and so on^1^. According to World Health Statistics in 2021, CVD remains the leading cause of death from NCDs, particularly in low-income developing countries^2^. So how to effectively maintain normal cholesterol levels is an important public health issue. Statins and fibrates are the most commonly used cholesterol-lowering drugs, but long-term use can cause serious side effects, including increasing the risk of rhabdomyolysis, allergic syndrome, and cognitive impairment^3^.

In recent years, the cholesterol-lowering effect of probiotics has attracted the attention of researchers^4^. Compared with drug therapy, its characteristics of high efficiency, safety and economy have been favored by consumers. Studies have shown that *Lactobacillus plantarum* NCU116 can also effectively regulate lipid metabolism in hyperlipidemia rats, enough to reduce total cholesterol (TC) levels in hyperlipidemia rats, and has the potential to regulate lipid metabolism, liver and adipose tissue morphology^5^. Both live and ultrasound-inactivated *Lacticaseibacillus casei* were able to reduce TC levels and control insulin resistance in male rats fed a high-fat diet^6^. Probiotics can reduce cholesterol by the regulating gut microbiota and its metabolites^7^. The change of intestinal microbiota, which is known as the “second brain” of the human body, may affect the energy homeostasis, systemic inflammation, lipid metabolism, glucose balance, and insulin sensitivity of the host^8^. In addition, the richness and diversity of gut microbiota, especially the ratio of *Firmicutes* to *Bacteroidetes* (F/B ratio)^9^, can be significantly improved by taking probiotics. Use of fecal microbiota transplantation (FMT) has shown that regulating intestinal microbiota can effectively treat metabolic syndrome^10^.

At present, many probiotic products have been developed to regulate cholesterol, but all of them focus on living bacteria to prevent cholesterol from rising. Furthermore, some studies found that dead bacteria and their metabolites, called “postbiotics“^11,12^, can still have physiological activity^13^. Previously, our team identified a probiotic strain *Lactobacillus plantarum* H6 (*L.p* H6, CGMCC 18205, Patent No. ZL 201910955071. X) with good tolerance in the digestive tract. The strain H6 has been developed into a commodity. The in vitro cholesterol clearance ability of H6 reached 92.07%, and animal testing has shown that it may effectively improve intestinal microbiota dysbiosis and liver damage caused by a high cholesterol diet^14^. In this study, the regulation effect of viable, inactivated and ultrasonically-lysed *L.p* H6 on cholesterol and its influence on intestinal microbiota were evaluated, and the therapeutic effects of *L.p H6* on hypercholesterolemic mice were systematically studied, which provided a theoretical basis for the industrial development of probiotics and the treatment of cholesterol diseases.

## Results

### Effects of v/i/uH6 on serum and liver physicochemical indexes of mice

**Fig. 1:**
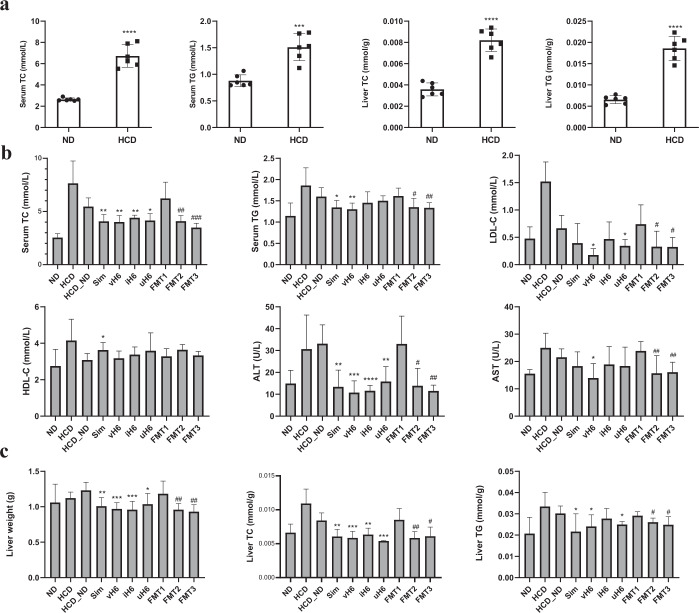
Effects of v/i/uH6 and FMT on serum and liver physicochemical indices in mice. **a** Serum TC and TG and liver TC and TG levels in hypercholesterolemia model mice. Data are represented as the mean ± SEM (*n* = 6). ***P < 0.001, ****P < 0.0001 vs. control ND. **b** Serum TC, TG, LDL-C, high-density lipoprotein cholesterol (HDL-C), ALT, and AST levels after 12 weeks. **c** Liver weight, TC, and TG levels. Data are represented as the mean ± SEM (*n* = 8). *P < 0.05, **P < 0.01, ***P < 0.001, ****P < 0.0001 vs. control HCD_ND; ^#^*P* < 0.05, ^##^*P* < 0.01, ^###^*P* < 0.001 vs. control FMT1. HCD_ND refers to the group of hypercholesterolemia model mice; v/i/uH6 refers to viable (vH6), heat-inactivated (iH6), and ultrasonically-lysed (uH6) bacteria cells; FMT1/2/3 refers to using feces from HCD_ND, Sim, and vH6 groups, respectively.

Compared with the ND group, the HCD group had no significant differences in food intake and weight gain (Supplementary fig. 1a), while, TC and Triglyceride (TG)in serum and liver of mice in the HCD group increased significantly, and the content was ~2–3 times that of the ND group (Fig. 1a), indicating that the hypercholesterolemia mice model has been successfully established.

Compared with the HCD_ND group, after 12 weeks of treatment with v/i/uH6 and FMT3, the physicochemical indexes related to lipid metabolism in mice serum were improved (Fig. 1b), TC was significantly decreased, especially the serum TC in the FMT3 group, which decreased by 43.98% (*P* < 0.05). The serum TG levels of mice given vH6 and FMT3 were significantly decreased by 18.69% and 17.17%, respectively (*P* < 0.05). Compared with the HCD group, low-density lipoprotein cholesterol (LDL-C) levels were significantly lower when given vH6, uH6 and FMT3 (0.18–0.34 mmol/L vs 1.5 mmoL). Moreover, v/i/uH6 and FMT3 significantly reduced the serum alanine aminotransferase (ALT) levels of mice, especially those given vH6 and iH6 (10.76 U/L and 11.58 U/L, respectively). vH6 and FMT3 significantly decreased serum aspartate aminotransferase (AST) levels (*P* < 0.05).

v/i/uH6 and FMT3 similarly improved the liver weight, TC and TG (except for iH6) contents of mice (Fig. 1c). For example, when given vH6 and iH6, liver weight decreased by 21.27% and 22.10%, respectively (*P* < 0.05). v/i/uH6 decreased TC by 30.44%, 24.49%, and 35.67%, respectively (*P* < 0.05). Overall, v/i/uH6 and FMT3 improved serum and liver physicochemical properties, including lower TC, TG, LDL-C, ALT, AST in the serum, and TC, TG in the liver at various levels.

### Effect of v/i/uH6 on liver degeneration in hypercholesterolemic mice

**Fig. 2:**
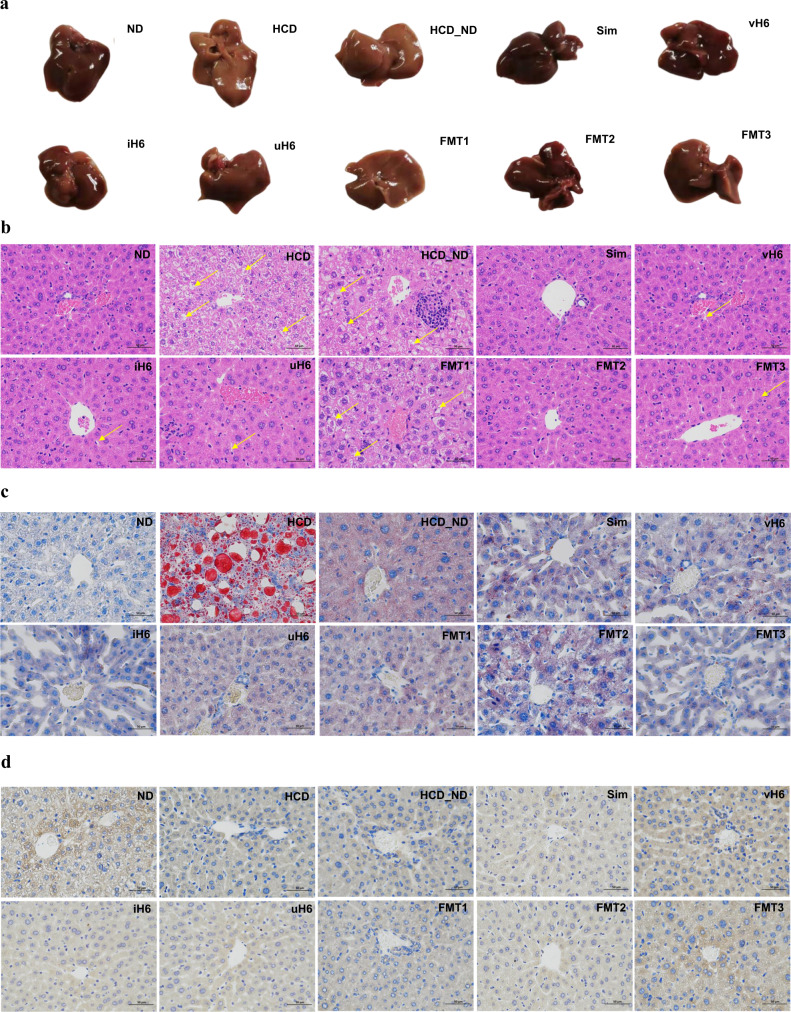
Effects of v/i/uH6 and FMT on liver degeneration in hypercholesterolemia mice. **a** Images of mice livers after different treatments. **b**, **c** Representative images of H/E staining and Oil Red O staining of liver sections (400×, scale bar = 50 μm). Some local damaged portions of hepatocyte are indicated by yellow arrows in H/E staining. **d** Representative images of immunofluorescent staining of liver sections using anti-UCP1 (400×, scale bar = 50 μm). HCD_ND refers to the group of hypercholesterolemia model mice; v/i/uH6 refers to viable (vH6), heat-inactivated (iH6), and ultrasonically-lysed (uH6) bacteria cells; FMT1/2/3 refers to using feces from HCD_ND, Sim and vH6 groups, respectively.

Mice fed with HCD showed hepatomegaly with a grayish-yellow hue. However, v/i/uH6 and FMT3 inhibited hepatomegaly, the color (reddish-brown) and soft texture of liver were close to normal. (Fig. 2a). HE staining showed that in the HCD group, the arrangement of liver cells was loose and disordered, a large number of fat vacuoles of different sizes and numbers appeared, the liver cells were enlarged, partially necrotic, and the liver cells were severely damaged. (Fig. 2b). When mice were treated with v/i/uH6 and FMT3, the damage of liver cells was obviously reduced, the fat droplets in the cytoplasm were reduced, and the liver cells were arranged neatly.

Oil red O staining showed that liver lipid droplets in hepatocytes of HCD mice showed particle accumulation and fusion into flakes (Fig. 2c). However, when mice were treated with vH6, iH6 and FMT3, the lipid droplets were significantly less, the size was smaller, and the distribution was irregular and uneven.

The expression of UCP1 in liver tissues was detected by immunohistochemical staining (Fig. 2d). Compared with HCD_ND and FMT1, UCP1 expression was significantly enhanced in mice given vH6, iH6 and FMT3, and there was a large area of positive brown particles in the liver tissues. In conclusion, v/i/u H6 and FMT3 can be observed to improve liver degeneration to varying degrees by staining liver sections.

### Effect of v/i/uH6 on glucose tolerance test and insulin tolerance test in hypercholesterolemic mice

**Fig. 3:**
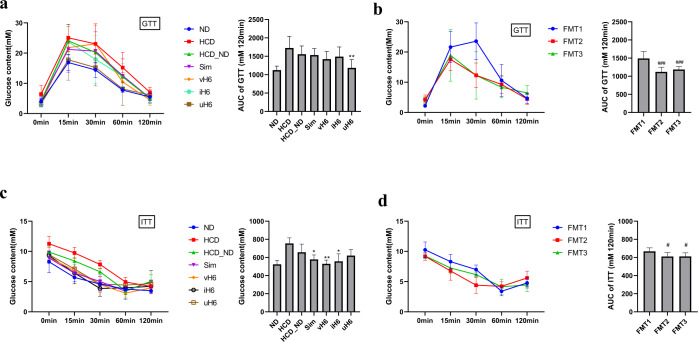
Effects of v/i/uH6 and FMT on glucose tolerance test (GTT) and insulin resistance test (ITT) in hypercholesterolemic mice. **a**, **b** GTT: mice fasted overnight were injected with glucose (2 mg/kg) intraperitoneally. Blood glucose was determined at 0, 15, 30, 60, and 120 min then. **c**, **d** ITT: mice fasted for 4 h were injected with insulin (0.75 U/kg). Blood glucose was determined at 0, 15, 30, 60, and 120 min then. AUC value was calculated. Data are presented as the mean ± SEM (*n* = 8). *P < 0.05, **P < 0.01vs. control HCD_ND; ^#^*P* < 0.05, ^##^*P* < 0.01, ^###^*P* < 0.001 vs. control FMT1. HCD_ND refers to the group of hypercholesterolemia model mice; v/i/uH6 refers to viable (vH6), heat-inactivated (iH6), and ultrasonically-lysed (uH6) bacteria cells; FMT1/2/3 refers to using feces from HCD_ND, Sim and vH6 groups, respectively.

In the HCD group, the glucose levels of mice were disturbed and the blood glucose levels increased sharply. v/i/uH6 and FMT3 decreased the area under curve (AUC) values corresponding to glucose tolerance in mice, which were significantly decreased by 23.89% and 20.50% in the uH6 and FMT3 groups, respectively (*P* < 0.05, Fig. 3a, b). Similarly, v/i/uH6 and FMT3 improved insulin tolerance of mice, as evidenced by reduced glucose and lower AUC values in the insulin tolerance test, which were a significantly decreased by 19.53%, 15.10%, and 8.35% in the vH6, iH6, and FMT3 groups, respectively (*P* < 0.05, Fig. 3c, d). These results indicate that v/i/uH6 and FMT3 can improve glucose and insulin tolerance in hypercholesterolemic mice.

### Effect of v/i/uH6 on intestinal microbiota structure of hypercholesterolemic mice

**Fig. 4:**
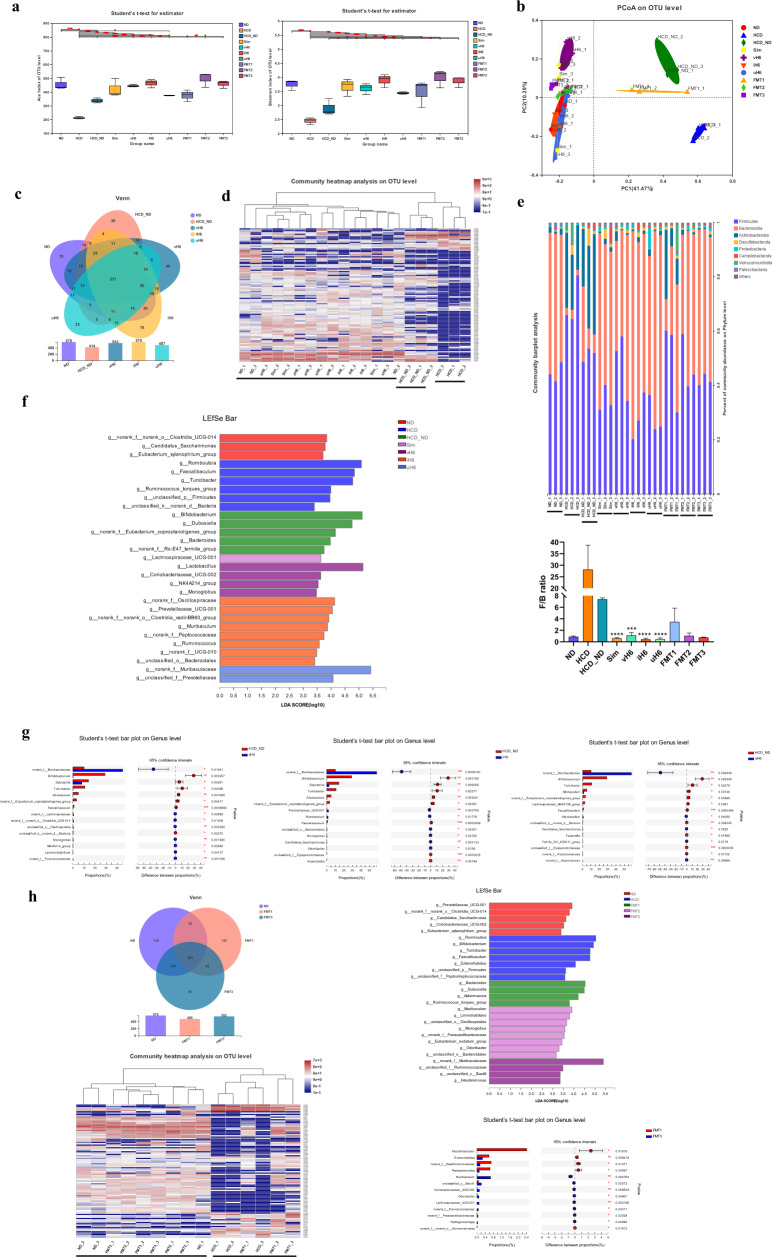
Effects of v/i/uH6 and FMT on intestinal microflora structure in hypercholesterolemic mice. **a** Alpha-diversity assessed by richness (ACE) and diversity (Shannon). **b** Beta diversity calculated using weighted UniFrac by PCoA. **c** Venn diagram of OTUs in mice given v/i/uH6. **d** Heatmap of the relative abundance of the top 100 OTUs in mice given v/i/uH6. **e** Relative abundance of microbiota at the phylum level and *Firmicutes*/*Bacteroidetes* ratio. **f** LDA score (LDA > 2.5) in mice given v/i/uH6. **g** Two groups comparison using a Student’s *t* test. **h** Venn diagram of OTUs, heatmap of the relative abundance of the top 100 OTUs, LDA score and two groups comparison using a Student’s *t* test in fecal microbiota transplantation groups. Data are presented as the mean ± SEM (*n* = 3). *P < 0.05, **P < 0.01, ***P < 0.001. HCD_ND refers to the group of hypercholesterolemia model mice; v/i/uH6 refers to viable (vH6), heat-inactivated (iH6), and ultrasonically-lysed (uH6) bacteria cells; FMT1/2/3 refers to using feces from HCD_ND, Sim and vH6 groups, respectively.

A total of 1061 OTUs, including 18 phyla, 33 classes, 78 orders, 112 families, 196 genera, and 305 species, were identified in mice intestinal microbiota by 16S rRNA sequencing technology. The α diversity of intestinal microbiota of different treated mice was evaluated based on OTU levels. The Ace, Shannon, and Shannoneven indexes of the intestinal microbiota of mice given v/i/uH6 and FMT3 were significantly higher than those of the HCD_ND and FMT1 groups (Fig. 4a and Supplementary fig. 2b). The β-diversity principal coordinate analysis (PCoA) showed that the intestinal microbiota of mice given v/i/uH6 and FMT3 groups was similar to that of the ND group but significantly different from that of the HCD, HCD_ND and FMT1 groups (Fig. 4b). Compared to HCD_ND, v/i/uH6 increased the abundance of intestinal microbiota in mice, and the number of OTUs increased by 31.16%, 39.86%, and 17.63%, respectively (Fig. 4c). Heat maps based on OTU levels showed that the intestinal microflora structure of the HCD, HCD_ND groups and other treatment groups were clearly divided into three parts, and the community abundance of v/i/uH6 was more similar to that of the ND and Sim groups (Fig. 4d). Further analysis of intestinal microbiota composition at the phylum level (Fig. 4e) showed that in HCD group, the *Firmicutes* increased and the *Bacteroidota* decreased in the intestine, which led to an increase of F/B ratio. While v/i/uH6 reduced the relative abundance of *Firmicutes* by 5.94%, 44.96%, and 44.64% respectively, it increased the relative abundance of *Bacteroidota* by 234.85%, 377.05%, and 362.53% respectively, and reduced the F/B ratio significantly compared to the HCD_ND group.

LEfSe analysis showed significant differences in intestinal microbiota structure between the different treatment groups (Supplementary fig. 2f), and linear discriminant analysis (LDA) was used to evaluate the predominant bacteria in the different groups. The dominant bacteria of mice given vH6 were *Lactobacillus*, *Coriobacteriaceae_UCG-002*, and *norank_f__Oscillospiraceae*, *Muribaculum*, *unclassified_o__Bacteroidales*, and *Ruminococcus* when given iH6, and *norank_f__Muribaculaceae* when given uH6 (Fig. 4f). Student’s *t* test showed that in the HCD_ND group, the abundance of *Faecalibaculum*, *Allobaculum* and *Turicibacter* increased significantly compared to that of the v/i/uH6 treated group in which the abundance of *norank_f__Muribaculaceae* increased significantly compared to that of the HCD_ND group (Fig. 4g). In addition, compared to the HCD_ND group, i/uH6 increased *unclassified_o__Oscillospirales* and *Lachnoclostridium*, and vH6 increased *Muribaculum* significantly, so these dominant bacteria could be potential biomarkers for effective cholesterol reduction by *L.p*.

Fecal microbiota transplantation (FMT) using feces from mice treated by vH6 also improved the intestinal microbial composition induced by HCD (Fig. 4h). Compared to the FMT1 treatment, the number of intestinal microbes OTUs of FMT3 treated mice increased by 15%, and the heat map of community distribution was more similar to that of the normal and FMT2 treated mice. Similarly, the amount of *Firmicutes* decreased by 15.45% and that of *Bacteroidota* increased by 79.3% in FMT3 treated mice. The dominant bacteria of FMT3 treated mice were *norank_f__Muribaculaceae*, *unclassified_f__Ruminococcaceae*, *unclassified_c__Bacilli*, and *Intestinimonas*, of which *norank_f__Muribaculaceae* was also the most abundant dominant bacteria of uH6 treated mice. Compared with FMT1 treatment, FMT3 increased the abundance of *Enterorhabdus*, *Muribaculum*, *unclassified_c__Bacilli*, *Coriobacteriaceae_UCG-002* and *norank_f__Paracaedibacteraceae* increased. In addition, FMT3 decreased *Faecalibaculum* levels, which was consistent with the v/i/uH6 treated group. These results indicated that v/i/uH6 and FMT3 can alleviate the dysbiosis of the intestinal microbiota of hypercholesterolemic mice by regulating intestinal microbes.

### Effect of v/i/uH6 on fecal metabolites in hypercholesterolemic mice

**Fig. 5:**
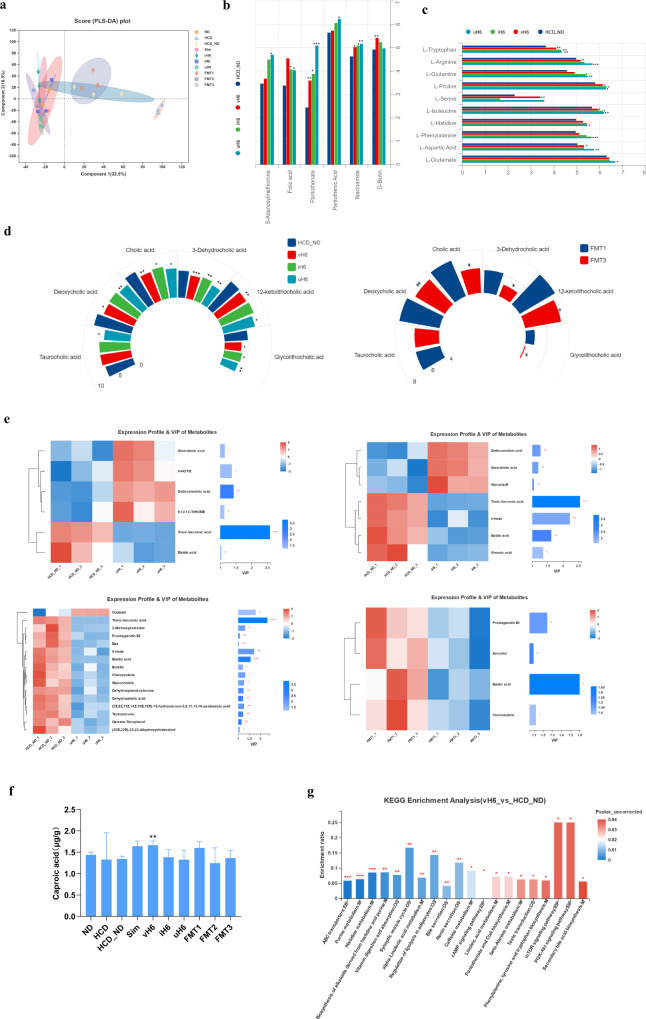
Effects of v/i/uH6 and FMT on intestinal metabolites in hypercholesterolemia mice. **a** Partial least squares-discriminant analysis (PLS-DA) based on the microbial metabolites. **b**–**d** The relative content of vitamins-cofactors, amino acids and bile acids detected by UPLC/Q-TOF-MS/MS based non-targeted metabolomics approach. **e** The clustering heatmap and VIP bar map of the differential lipids were compared between the two groups. **f** The content of caproic acid. **g** KEGG pathway enrichment analysis of differential metabolites in HCD_ND vs. vH6. Data are presented as the mean ± SEM (*n* = 3). *P < 0.05, **P < 0.01, ***P < 0.001. HCD_ND refers to the group of hypercholesterolemia model mice; v/i/uH6 refers to viable (vH6), heat-inactivated (iH6), and ultrasonically-lysed (uH6) bacteria cells; FMT1/2/3 refers to using feces from HCD_ND, Sim and vH6 groups, respectively.

Non-targeted detection of mice fecal metabolites was conducted based on UPLC/Q-TOF-MS/MS. MS and MS/MS information was matched with the metabolic public databases HMDB (http://www.hmdb.ca/) and Metlin (https://metlin.scripps.edu/). A total of 1114 fecal metabolites were identified. PLS-DA analysis showed that the groups of HCD, HCD_ND, and FMT3 were obviously separated from the other treatment groups (Fig. 5a), and the substitution test model showed that the PLS-DA model was reliable (Supplementary fig. 3a). According to the heat map, the fecal metabolites of mice given v/i/uH6 and FMT3 were more similar to the ND group, but were obviously separated from the HCD, HCD_ND, and FMT1 groups (Supplementary fig. 3b). All metabolites were classified as vitamins-cofactors, amino acids, bile acids and lipids. Compared with the HCD_ND group, v/i/uH6 increased relative content of six vitamins-cofactors, among which pantothenate and niacinamide were significantly increased (Fig. 5b). However, FMT3 did not change the level of vitamins-cofactors significantly (Supplementary figure 3c). Compared to HCD_ND group, v/i/uH6 significantly increased the content of amino acids (Fig. 5c), such as l-Tryptophan, l-Proline, l-Phenylalanine, and l-Glutamate, and the content of branched-chain amino acids (BCAA), such as l-Isoleucine. In terms of bile acids, compared to the HCD_ND group, v/i/uH6 reduced the content of primary bile acids by 18.10%, 13.31%, and 21.00% respectively, and reduced the content of secondary bile acids, such as Deoxycholic acid, 3-Dehydrocholic acid, 12-ketolithocholic acid, Glycolithocholic acid. FMT3 had the same trend in bile acids regulation (Fig. 5d). The differential metabolites between different treatment groups were screened by a cluster heat map and VIP bar graph (Fig. 5e). v/i/uH6 and FMT3 treated mice were identified for 7, 7, 16, and 4 notable lipid metabolites, respectively. Compared to the HCD_ND group, vH6 significantly upregulated four lipids metalolites, namely 9,12,13-TriHOME, 9-HOTrE, stearidonic acid and dodecanedioic acid. iH6 significantly upregulated stearidonic acid, dodecanedioic acid and alprostadil. uH6 significantly upregulated ouabain one. FMT3 reduced all differential metabolites compared to FMT1. On the whole, there are similarities in the differential metabolites of v/i/uH6 and FMT3, such as elaidic acid, trans-vaccenic acid, stearidonic acid, etc.

For targeted detection of SCFAs, a Thermo TRACE1310-ISQ LT gas chromatograph equipped with an Agilent HP-INNOWAX capillary column was used. Compared to the HCD_ND group (Fig. 5f), vH6 increased the content of caproic acid (*P* < 0.05), acetic acid, propionic acid, isobutyric acid, butyric acid, valeric acid, and total SCFAs; uH6 increased the content of acetic acid, propionic acid and valeric acid. Compared to the FMT1 group FMT3 increased the content of Acetic acid and Propionic acid, although none of these differences were statistically significant (Supplementary fig. 3e).

The biological processes of fecal metabolites were predicted using KEGG pathway analysis. Analysis of differential metabolites between vH6 and HCD_ND groups revealed that vH6 regulated hypercholesterolemia in mice by many pathways, including vitamin digestion and absorption, lipolysis regulation, cAMP signaling pathway, linoleic acid metabolism, phenylalanine, tyrosine and tryptophan biosynthesis, mTOR signaling pathway, PI3K-Akt signaling pathway, and secondary bile acid biosynthesis. Similar to vH6, iH6, and uH6 are involved in regulating metabolic pathways, such as the cAMP signaling pathway, bile secretion, lipolysis regulation, or mTOR signaling pathway (Fig. 5g and Supplementary fig. 3f). Relative to FMT1, FMT3 may regulate metabolism via bile secretion and secondary bile acid biosynthesis. In conclusion, v/i/uH6 all improved intestinal metabolites in hypercholesterolemia mice to different degrees. In particular, the v/i/uH6 cells increased the intestinal flora metabolism of vitamins-cofactors, as well as amino acids, while decreasing the relative content of primary bile acids.

### Correlation of microbial community, physicochemical indicators, and metabolites in v/i/uH6 treated mice

**Fig. 6:**
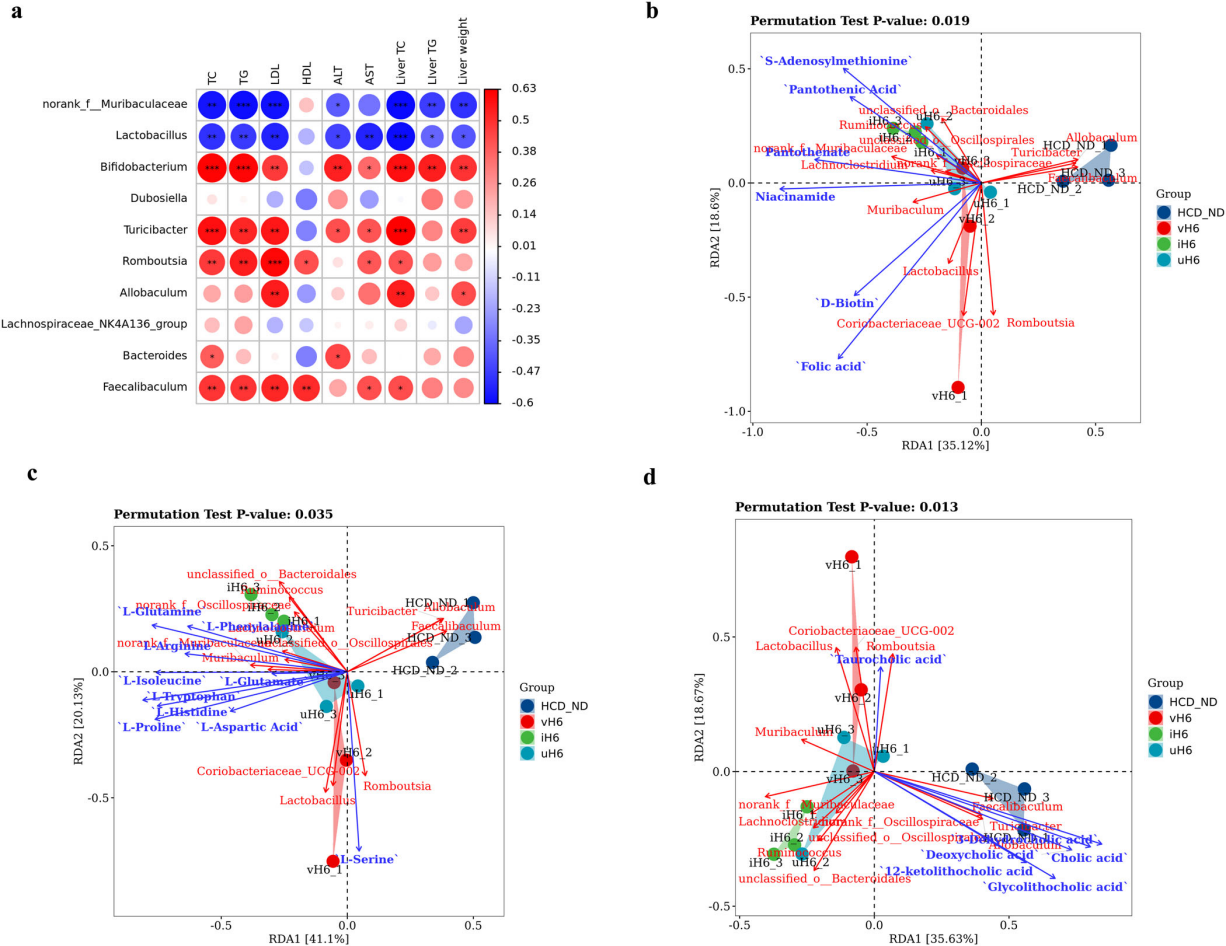
The correlation analysis of physicochemical indexes and metabolites of mice given v/i/uH6. **a** Correlation analysis between top 10 richest bacteria and physicochemical indices of hypercholesterolemia by Spearman method. **b**–**d** Redundancy analysis (RDA) of the correlation between v/i/uH6 microbial community and fecal metabolites (vitamins-cofactors, amino acids, bile acids). Data are presented as the mean ± SEM (*n* = 3). *P < 0.05, **P < 0.01, ***P < 0.001. HCD_ND refers to the group of hypercholesterolemia model mice; v/i/uH6 refers to viable (vH6), heat-inactivated (iH6), and ultrasonically lysed (uH6) bacteria cells.

Further analysis of Pearson correlations between gut microbiota (top 10 genera at the genus level) and physicochemical indicators of hypercholesterolemia showed that *norank_f__Muribaculaceae* and *Lactobacillus* had significant negative correlations with TC, TG, LDL, ALT, AST, and liver weight. *Turicibacter*, *Romboutsia*, and *Faecalibaculum* all had significant positive correlations with these indices (Fig. 6a). *Allobaculum* also had a significant positive relationship with LDL, liver TC and liver weight. The correlation between fecal metabolites and microbial community structure was assessed by redundancy analysis (RDA), as shown in Fig. 6b–d and Supplementary fig. 4a, b. In the v/i/uH6 treated group, *Faecalibaculum*, *Allobaculum*, and *Turicibacter* showed a significant negative relationship with other bacteria, confirming previous findings. D-biotin and folic acid had a strong positive correlation with *Lactobacillus*, *Coriobacteriaceae_UCG-002* and *Muribaculum*, while vitamins-cofactors had a positive correlation with the dominant bacteria of v/i/uH6 treated mice (Fig. 6b). l-serine correlated strongly with *Lactobacillus* and *Coriobacteriaceae_UCG-002*, while the remaining amino acids correlated with v/i/uH6 dominant bacteria (Fig. 6c). For bile acids, glycolithocholic acid had no correlation with *unclassified_o__Bacteroidales* and *Turicibacter*. *Romboutsia* and *Faecalibaculum* showed significant positive correlations with most bile acids (Fig. 6d). These results indicated a strong correlation between the production and abundance of fecal metabolites and intestinal microbes in hypercholesterolemic mice given v/i/uH6. Similarly, in the FMT3 group, a correlation between microbial communities and physicochemical indicators and metabolites was discovered, but there was no statistical significance (Supplementary fig. 4c–g). In brief, the Pearson correlation analysis showed that *norank_f__Muribaculaceae* and *Lactobacillus* had a negative correlation with blood lipid levels. RDA indicated a strong positive correlation between the production and abundance of fecal metabolites and intestinal microbes in hypercholesterolemic mice given v/i/uH6.

## Discussion

*Lactobacillus plantarum* H6 (CGMCC 18205) is a probiotic strain with a cholesterol-lowering effect that has been isolated from the fermented dough in our laboratory and has been granted a national invention patent (No. ZL 201910955071. X). Previously, we conducted in vivo experiments to assess the preventive effect of *L.p* H6 on hypercholesterolemia. The current study further evaluated its therapeutic effects on hypercholesterolemia. The dead and lysed H6 cells were also evaluated together in order to gain a more comprehensive understanding of H6’s regulatory effects on lipid metabolism.

The hypercholesterolemic mice given both viable and inactivated H6 increased their food intake, but had no significant change in weight gain. This finding is consistent with a previous study that found *Lactobacillus fermentum* ZJUIDS06 and *Lactobacillus plantarum* ZY08 had no effect on weight loss in hypercholesterolemic rats^15^. However, v/i/uH6 decreased liver weight significantly, as did stool from intestine of vH6 treated mice (FMT3), implying that v/i/uH6 could improve gut microbial function and thus enhance lipid metabolism and energy expenditure.

In clinical practice, the levels of TC, TG, LDL-C, ALT, and AST in the body are critical for health monitoring^16,17^. In this study, the strain H6, including live and dead cells, had good lipid-lowering effects, which was confirmed by biochemical indicators in the serum and liver, as well as histopathology test. Generally, cell viability is an important requirement for probiotics to perform their health-promoting functions^16^. However, postbiotics, which are non-viable bacterial cells, bacterial fractions, or cell lysates, might also provide physiological advantages to the host by offering extra bioactivity^6^. Our results showed that the heat-killed and ultrasonically-lysed H6 cells still maintain some physiological activity to a certain extent, which may be due to the ability of the cell wall components to adsorb cholesterol. Cholesterol has been shown to attach to cell wall peptidoglycan, which contains amino acids with binding capacity, but the exact mechanisms remain unknown^6^. In addition, exopolysaccharide has also been proven to improve lipid metabolism. It changes cholesterol homeostasis through the synthesis and combination of cholesterol and bile acids, and the specific mechanism still needs to be explored^18^.

Hepatic uncoupling protein 1(UCP1) has been shown to regulate extracellular succinate and inflammation in the liver by increasing energy expenditure and reducing hepatic lipids^19^. In this study, UCP1 increased in the liver of mice given v/i/uH6 and FMT3, indicating that the strain H6 inhibits liver degeneration in hypercholesterolemic mice via improving thermogenesis and lipid metabolism. In terms of glucolipid metabolism, both viable and dead H6 improved insulin tolerance, while lysed H6 improved glucose tolerance in mice. These findings are consistent with previous findings that found sterilized *Bifidobacteria* could improve glucose tolerance and insulin resistance, and thus lower blood glucose levels in mice with disorders of carbohydrate and lipid metabolism^20^. Of note, FMT3 using stool from mice given viable H6 has the same physiological benefits as the viable strains, such as lowering lipids and improving glucolipid metabolism, implying that viable H6 induced the production of beneficial metabolites by intestinal microorganisms in mice^21^.

It has been recognized that probiotic supplements leads to obvious changes in the intestinal microbial composition, and thus regulates dietary fat metabolism^22^. Therefore, gut microorganisms relevant to host health have been isolated and identified. The role of intestinal microorganisms in health or disease is now being recognized^23^.

In this study, both v/i/uH6 and FMT3 were sufficient to improve microbial composition in the intestinal induced by a high cholesterol diet. *Lactobacillus* and *Coriobacteriaceae_UCG-002* were enriched in mice given vH6, which is consistent with the previous findings in our laboratory^14^. It was found that *Lactobacillus* was a common beneficial bacterium with the ability to regulate cholesterol metabolism due to its high bile salt hydrolase activity^24,25^. Four dominant genera were found in mice given v/i/uH6, including *norank_f__Oscillospiraceae*, *Muribaculu*, *unclassified_o__Bacteroidales*, and *Ruminococcus*, among which, *Ruminococcus* has been found enriched in the intestinal tract of children with normal weight, while less *Oscillospiraceae* were detected in obese individuals, suggesting that these two genera negatively correlated with cholesterol-lowering^26^. Both uH6 and FMT3 increased *norank_f__Muribaculaceae*, a well-defined anti-inflammatory bacteria.

*Muribaculaceae* were named after the analysis and description of the problems and characteristics of the family S24-7 of bacteria in terms of diversity, ecology, functional potential, and germline occurrence^27^. It is functionally different from its neighboring family and multifunctional with respect to complex carbohydrate and high calorie degradation^28^. Studies have shown that the community of *Muribaculaceae* in the intestinal flora of high fat diet mice is significantly reduced^29^. *Muribaculacea* is the most abundant bacterium in the intestinal at genus level in our study, relative to mice fed with high cholesterol diet, *Muribaculacea* was remarkably higher in mice give i/uH6 or FMT3, indicating it may have an important role in the lipid metabolism. Besides, FMT3 also increased *unclassified_f__Ruminococcaceae*, *unclassified_c__Bacilli*, and *Intestinimonas. Intestinimonas* was reported to control body weight and prevent type II diabetes in a cohort study of health and metabolic disease samples^30^. *Allobaculum and Turicibacter* were genera that could induce inflammation and obesity^26,31^. *Allobaculum* and *Faecalitalea* were found to be more abundant in mice treated with pork proteins and lard, suggesting these genera were positively correlated with inflammation^32^. Similar to these findings, we found that the abundance of *Faecalibaculum*, *Allobaculum* and *Turicibacter* increased in mice fed with high cholesterol diets, while decreasing in mice given v/i/uH6. Although *Bifidobacterium* is generally recognized as a beneficial bacterium^33,34^, it was found to be less abundant in mice given v/i/uH6 or FMT3 in this study, and the reasons for this need to be further investigated.

Many nutrients are known to play a role in the regulation of lipid metabolism^35,36^. A vitamin-rich diet may reduce the risks of coronary heart disease and atherosclerosis, as B-complex vitamins have been shown to lower TC and TG levels in the body. Niacin, a B-complex vitamin, is used to treat atherosclerotic disease in combination with some lipid-lowering drugs^37^. α-methyl-l-tryptophan could improve hyperglycemia, insulin resistance and hepatic steatosis^38^, while l-glutamic acid could inhibit the progression of atherosclerosis and fatty liver disease^39^. In this study, v/i/uH6 treatment increased the levels of vitamins-cofactors and amino acids related to the lipid metabolism of intestinal microbiota, partly explaining the mechanism of cholesterol-lowering action of the strain *L.p* H6. Bile acids (BAs) are important signaling molecules closely related to cholesterol metabolism. Many studies have shown that biotransformation of primary BAs by intestinal microorganisms can reduce fat accumulation in the liver and reduce blood lipid levels^40^. Our previous study showed that H6 could effectively exert their cholesterol-lowering effects through bile salt hydrolase as well as membrane adsorption, coprecipitation, and inhibition of cholesterol micelles. In addition, by promoting the expression of CYP7A1 gene and inhibiting the farnesoid X receptor pathway, viable H6 could increase the synthesis of BAs and the abundance of bacteria containing bile salt hydrolase (BSH) activity^14^. In this study, we also discovered that the a/i/sH6 and FMT3 reduced the relative levels of primary BAs, suggesting that H6 could prevent the absorption of BAs in the small intestine. SCFAs are another important metabolite of the intestinal microbiota, that has been shown to play an important role in the prevention and treatment of many diseases^41^. However, SCFAs amounts did not change after v/i/uH6 consumption in this study, which is in accordance with a recent randomized, double-blind trial showing that volunteers consuming a kind of probiotic *L. plantarum* Dad-13 effectively improved the lipid profile with no significant change in the concentration of SCFAs in the intestine^42^. The reason is not clear.

*Muribaculaceae* has been shown to have a negative correlation with mouse body weight and biochemical parameters^43^, from the observations recorded above, we investigated the correlation between gut microbiota and physicochemical parameters by Pearson correlation analysis and RDA, and we discovered that they were highly correlated. This correlation, however, needs to be validated at the strain level.

**Fig. 7:**
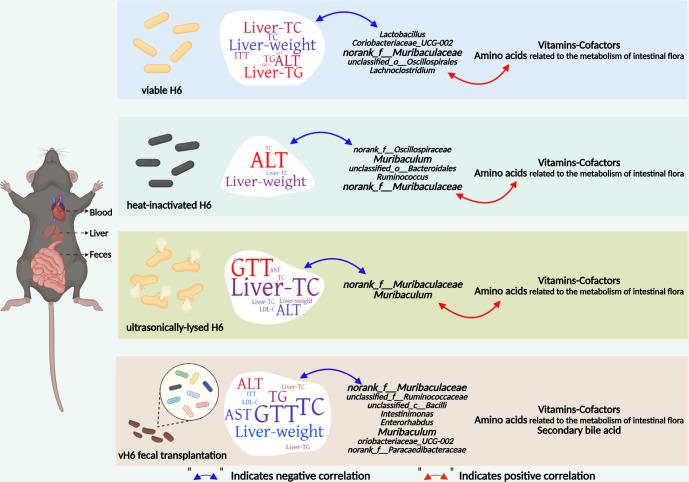
Schematic illustration of outlining the hypocholesterolemic effect of *L.p* H6 by regulating the intestinal microbiota and microbial metabolites related to lipid metabolism. v/i/uH6 cells could lower TC, TG, LDL-C, ALT, AST in the serum, and TC, TG in the liver at various levels, and improve GTT and ITT indexes. Also, the recovery of these biochemical indices and gut microbiome was found following FMT using stool from vH6 treated mice. *Muribaculaceae* was found to be the most abundant in the intestine of mice after v/i/uH6 treatments, suggesting that it could be used as a potential biomarker for effectively lowering cholesterol. The v/i/uH6 cells increased the intestinal flora metabolism of vitamins-cofactors, as well as amino acids, while decreasing the relative content of primary bile acids. The Pearson correlation analysis showed that *norank_f__Muribaculaceae* and *Lactobacillus* had a negative correlation with blood lipid levels. RDA indicated a strong positive correlation between the production and abundance of fecal metabolites and intestinal microbes in hypercholesterolemic mice given v/i/uH6. Overall, v/i/uH6 cells were effective in improving hypercholesterolemia in mice, and this effect was attributed partly to the regulation of intestinal microbiota and metabolites related to lipid metabolism. v/i/uH6 refers to viable (vH6), heat-inactivated (iH6), and ultrasonically-lysed (uH6) bacteria cells. (Figure was created with Biorender.com.).

This study investigated the therapeutic effect of a new patented strain *Lactobacillus plantarum* H6 in different states (active, heat-inactivated and ultrasonically-lysed) on hypercholesterolemic mice. As shown in Fig. 7, the v/i/uH6 cells and stool from vH6 treated mice (FMT3) improved hypercholesterolemia in mice, and this effect was partly attributed to the regulation of intestinal microbiota and metabolites related to lipid metabolism. *Muribaculaceae* could be employed as a potential biomarker for effective cholesterol reduction. Heat-inactivated and ultrasonically-lysed *L.p* H6 could be a promising postbiotic for regulating cholesterol metabolism. Although *L.p* H6 in the three states can improve hypercholesterolemia mice to different degrees, its mechanism is still unknown, and the effector molecules and signaling pathways of *L.p* H6 in lowering cholesterol are still worthy of investigation in future studies.

## Methods

### Materials and reagents

Simvastatin was purchased from Shandong Lu’an Anti-Pharmaceutical Group Saite Co., Ltd, Shandong, China. Insulin injection was purchased from Jiangsu Wanbang Biochemical Pharmaceutical Group Co., Ltd. Blood glucose meter and blood glucose test paper (type GA-3) were purchased from Sanno Biosensing Co., Ltd, Jiangsu, China. TC, TG, LDL-C, HDL-C, ALT and AST detection kits were purchased from Jiancheng Bioengineering Research Institute (Nanjing, China).

### Preparation of v/i/uH6 bacteria cells

*Lactobacillus plantarum* H6 (CGMCC 18205) was selected from the sticky dough, a traditional Chinese fermented flour product. Before the experiment, the bacterial cells were activated twice. The activated cells were inoculated with MRS medium, and cultured in anaerobic conditions at 37 °C for 24 h. After culture, the bacterial suspension was washed twice with physiological saline, and the concentration of the suspension was adjusted to 1 × 10^9^ cfu/mL (vH6). Inactivated cells (iH6) were prepared from bacterial suspension in water bath at 90 °C for 30 min with minor changes to the previous approach^44^. The bacterial suspension was treated with ultrasonic cell pulverizer for 5 s and 60 min at 9 s intervals to prepare bacterial ultrasonic lysate (uH6) with minor changes to the previous approach^45^ and plate culture showed that no viable bacteria grew.

### Animals and experimental design

Eighty-eight male C57BL/6 mice (5 weeks old and weighing 18–19 g) were purchased from Shenyang Changsheng Biotechnology Co., Ltd. (approval number: SCXK (Liao) 2021-0001, Liaoning, China). All animal procedures were carried out in accordance with the Guidelines for the Care and Use of Laboratory Animals of Jilin Agricultural University, and approved by the Animal Ethics Committee at Jilin Agricultural University. Mice were housed at room temperature (25 ± 1 °C) under 12 h light/12 h dark cycles. After one week’s adaptation, mice were divided into 3 groups, including the normal diet (Beijing Co-operative Feeds Co., Ltd.) group (ND), the high cholesterol diet (High cholesterol Rat Food, Dietz Biotechnology Co., Ltd.) group (HCD), and the treatment group.

After 4 weeks of feeding, serum and liver of the ND and HCD mice were taken out after euthanasia to measure TC and TG levels. When the lipid levels of the HCD group were 2–3 times higher than that of the ND group, the hypercholesterolemia model was considered to be successful, and used in the following experiments. From the 5th week to the 12th week, all groups were fed the normal diet except HCD group, meanwhile, v/i/uH6 cells (1×10^9^ cfu/mL) and Simvastatin (3.80 mg/kg BW) were respectively gavaged daily. Group ND and HCD were gavaged with 0.9% NaCl in equal amount every day (HCD group was fed with a normal diet and was named HCD_ND group).

**Fig. 8:**
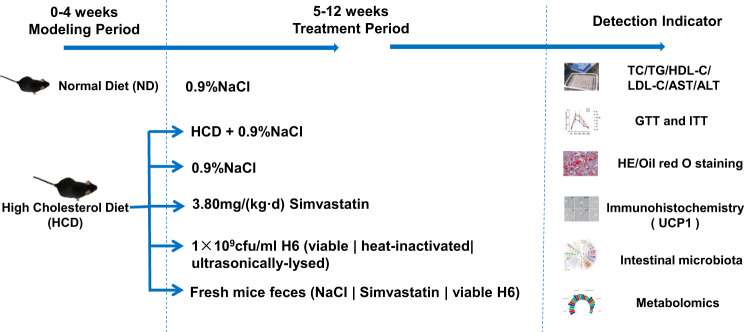
The flowchart of the experiment. In the first 4 weeks, 88 male C57BL/6 mice were randomly divided into a normal diet group (ND) and high cholesterol diet group (HCD). From the 5th week to the 12th week, all groups were fed the normal diet except HCD, meanwhile, v/i/uH6 cells (1 × 10^9^ cfu/mL) and Simvastatin (3.80 mg/kg BW) were gavaged daily. Group ND and HCD were gavaged with 0.9% NaCl in equal amount every day. The hypercholesterolemia model mice were used as recipients of the FMT test. The FMT was carried out using feces from mice treated with NaCl, and vH6, respectively. (Figure was originated by author. No third party materials were used).

The experimental design of fecal microbiota transplantation (FMT) test is as follows: under aseptic conditions, 300 mg of fresh feces were collected from the HCD_ND, Sim and vH6 groups respectively every day, re-suspended in 3 mL of sterile 0.9% NaCl, then centrifuged at 800 g for 3 min^46^. Then, the supernatant was collected and the hypercholesterolemia model mice (HCD_ND group) were used as recipients of the FMT test. FMT using feces from HCD_ND, Sim and vH6 groups are named FMT1, FMT2, and FMT3, respectively, as shown in Fig. 8. On the day before killing the mice, the feces of mice were collected under aseptic conditions, frozen in liquid nitrogen and kept at −80 °C. After 12 weeks of animal experiments, mice were anesthetized with ether and killed by neck separation. Blood was collected, centrifuged at 3000 g min at 4 for 15 °C to obtain serum, which was stored at −20 °C for later use. The liver was collected and weighed, part of it was fixed with 4% paraformaldehyde, and part of it was frozen in liquid nitrogen, and stored at −80 °C.

### Glucose and insulin tolerance test

At the 8th week of v/i/uH6 and FMT3 treatment, mice were fasted overnight (for glucose tolerance test) or for 4 h (for insulin tolerance test), and were injected intraperitoneally with glucose (2 g/kg BW) or insulin (0.75 U/kg BW), respectively. Tail blood was collected at 0, 15, 30, 60, and 120 min after injection, and the blood glucose was measured by blood glucometer^8^ according to manufacturer instructions. The AUC value of each group was calculated.

### Detection of biochemical markers in blood and liver

The contents of TC, TG, HDL-C, LDL-C, AST, and ALT in serum and TC, TG in liver were determined with the reagent kits from Nanjing Jiancheng Biomedical Company.

### Histopathology of liver

HE staining: dewaxed paraffin sections of the liver were stained in hematoxylin staining solution for 3–5 min, then washed in running water. After that, the tissue sections were dehydrated in 85% and 95% gradient alcohol for 5 min, dyed in eosin staining solution for 5 min, sealed with neutral gel, and ×400 images were collected under a microscope.

Oil red O staining: fresh frozen tissue sections were fixed, and then stained with Oil red O. The sections were removed, left for 3 s, and then dipped sequentially into two cups of 60% isopropanol for 3 s and 5 s each. Then the sections were re-stained with hematoxylin for 3–5 min, and were sealed with glycerol gelatin sealer, and ×400 images were collected under a microscope.

Immunohistochemical analysis: tissue sections were dewaxed for antigen repair, placed in 3% hydrogen peroxide solution to block endogenous peroxidase, sealed at room temperature for 30 min by dripping 3% BSA, incubated with primary antibody overnight at 4 °C, and incubated with secondary antibody for 50 min at room temperature. Then, the newly prepared DAB color solution was dripped dropwise, and the chromogenic time was controlled under a microscope. The nuclei was re-stained with hematoxylin for 3 min, and sealed with neutral gel for microscopic imaging at ×400 magnification.

### 16S rRNA sequencing

Microbial community genomic DNA was extracted from fecal samples using the E.Z.N.A.® soil DNA Kit (Omega Bio-tek, Norcross, GA, U.S.). The hypervariable region V3-V4 of the bacterial 16S rRNA gene was amplified with primer pairs 338F (5′-ACTCCTACGGGAGGCAGCAG-3’) and 806R (5′-GGACTACHVGGGTWTCTAAT-3′). The PCR product was extracted from 2% agarose gel, purified using the AxyPrep DNA Gel Extraction Kit (Axygen Biosciences, Union City, CA, USA), and quantified using Quantus™ Fluorometer (Promega, USA). The Miseq PE300/NovaSeq PE250 platform from Illumina was used for sequencing (Shanghai Majorbio Bio-pharm Technology Co., Ltd). The raw sequences were quality-controlled using fastp^47^ software (https://github.com/OpenGene/fastp, version 0.20.0) and FLASH^48^ software (http://www.cbcb.umd.edu/software/flash, version 1.2.7) for splicing. Using UPARSE software (http://drive5.com/uparse/, version 7.1), the sequences were OTU clustered and chimeras were removed based on 97% similarity. The data was analyzed on the Megisense cloud computing platform (http://cloud.majorbio.com/).

### Metabolomic profiling of fecal samples

Non-targeted metabolomics analysis based on ultra-performance liquid chromatography-tandem time of flight mass spectrometry (UPLC/Q-TOF-MS/MS; Shanghai Majorbio Bio-pharm Technology Co., Ltd). 50 mg fecal samples were placed in 400 μL of extraction solution (acetonitrile: methanol = 1:1) for extraction, and 10 μL of supernatant was taken to the machine for analysis. The mobile phases consisted of 0.1% formic acid in water (solvent A) and 0.1% formic acid in acetonitrile: isopropanol (1:1, v/v) (solvent B). The solvent gradient changed according to the following conditions: from 0 to 3 min, 95% (A): 5% (B) to 80% (A): 20% (B), from 3 to 9 min, 80% (A): 20% (B) to 5% (A): 95% (B), from 9 to 13 min, 5% (A): 95% (B) to 5% (A): 95% (B), from 13 to 13.1 min, 5% (A): 95% (B) to 95% (A): 5% (B), from 13.1 to 16 min, 95% (A): 5% (B) to 95% (A): 5% (B) for equilibrating the systems. The sample injection volume was 2 μL and the flow rate was set to 0.4 mL/min. The column temperature was maintained at 40 °C. The mass spectrometric data were collected using a Thermo UHPLC-Q Exactive Mass Spectrometer equipped with an electrospray ionization (ESI) source operating in either positive or negative ion mode. Mass spectra of these metabolic features were identified by using the accurate mass, MS/MS fragments spectra, and isotope ratio difference by searching in reliable biochemical databases like the Human metabolome database (HMDB) (http://www.hmdb.ca/) and Metlin database (https://metlin.scripps.edu/). Pre-processed data was analyzed on the Megisense cloud computing platform (http://cloud.majorbio.com/).

A Thermo TRACE1310-ISQ LT gas chromatograph equipped with an Agilent HP-INNOWAX capillary column was used to target short-chain fatty acids, including acetic acid, propionic acid, butyric acid, isobutyric acid, valeric acid, isovaleric acid, and capric acid (Suzhou Panomic Biopharmaceutical Technology Co., Ltd.). Briefly, mix 50 mg of fecal sample with 50 μL 15% phosphoric acid, 100 μL 125 μg/mL internal standard (isocaproic acid) solution and 400 μL ether and centrifuge at 4 °C for 10 min at 12,000×*g*. Chromatographic conditions^49^: split injection, injection volume 1 μL, split ratio 10:1. The temperature of the sample inlet, ion source and transmission line was 250 °C, 300 °C, and 250 °C, respectively. The programmed ramp-up temperature started at 90 °C, then ramped up to 120 °C at 10 °C/min, then ramped up to 150 °C at 5 °C/min, and finally ramped up to 250 °C at 25 °C/min for 2 min. The carrier gas was helium with a flow rate of 1.0 mL/min and electron energy of 70 eV^50^. The contents of SCFAs were calculated according to the calibration curves of each standard.

### Statistical analysis

Data were statistically analyzed and graphed using Graphpad Prism 8.0, Majorbio Cloud Platform and Genes Cloud. The significance of differences between the two groups was analyzed by *t* test. For comparisons between 3 or more conditions, one-way ANOVA followed by Dunnett’s or Tukey’s multiple comparison tests was used. A correlation analysis was performed using Pearson analysis. Data are presented as means ± (SD). *P* < 0.05 was considered statistically significant. All experiments were repeated at least three times.

## Supplementary information

Supplementary information

## Data Availability

The datasets presented in this study can be found in online repositories. The names of the repository/repositories and accession number(s) can be found below: https://www.ncbi.nlm.nih.gov/, PRJNA882947 for 16S rRNA sequencing, and http://www.ebi.ac.uk/metabolights/MTBLS5977, MTBLS5977 and http://www.ebi.ac.uk/metabolights/MTBLS5978, MTBLS5978 for non-targeted metabolomics and targeted metabolomics. The other data from the current study are available from the corresponding authors on reasonable request.
