# Supplementary material for: Regulation of viable/inactivated/lysed probiotic Lactobacillus plantarum H6 on intestinal microbiota and metabolites in hypercholesterolemic mice
Source: NPJ Sci Food. 2022 Oct 31;6:50. doi: 10.1038/s41538-022-00167-x (PMC9622822; doi:10.1038/s41538-022-00167-x)
Supplement: Supplementary file 1 — Supplementary information [file 41538_2022_167_MOESM1_ESM.pdf]

Supplementary figure 1. Effects of v/i/uH6 and FMT on food intake and body weight indices of mice during modeling and gavage. (a-b) Changes in food intake and body weight of mice in each group during the modeling period and treatment period. (c) Whole-body view of mice at the 12th week of treatment. Data are presented as the mean ± SEM (n=8). *P < 0.05vs. control HCD_ND. HCD_ND refers to the group of hypercholesterolemia model mice; v/i/uH6 refers to viable (vH6), heat-inactivated (iH6), and ultrasonically-lysed (uH6) bacteria cells; FMT1/2/3 refers to using feces from HCD_ND, Sim and vH6 groups, respectively.

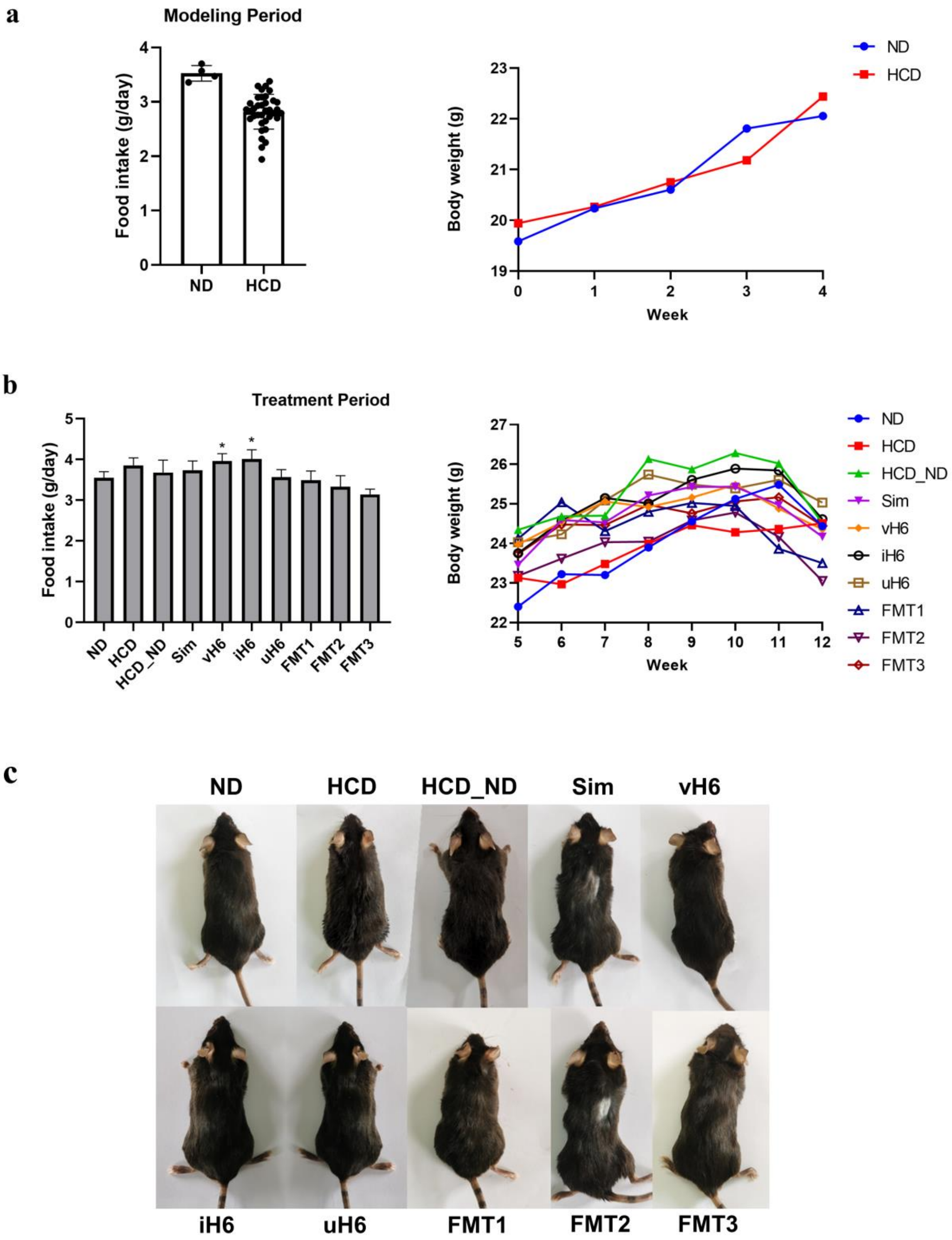
a
Modeling Period
Food intake (g/day)
ND
HCD
Body weight (g)
Week
ND
HCD
b
Treatment Period
Food intake (g/day)
ND
HCD
HCD_ND
Sim
vH6
iH6
uH6
FMT1
FMT2
FMT3
Body weight (g)
Week
ND
HCD
HCD_ND
Sim
vH6
iH6
uH6
FMT1
FMT2
FMT3
c
ND
HCD
HCD_ND
Sim
vH6
iH6
uH6
FMT1
FMT2
FMT3

Supplementary figure 2. Effects of v/i/uH6 and FMT on intestinal microflora structure in hypercholesterolemic mice. (a) ANOSIM (analysis of similarity) for sample grouping. (b) Shannoneven analysis of α diversity at the OUT level. (c) Dilatability curves of α diversity index Ace, Shannon, Shannoneven. (d) Rank-Abundance curves. (e) Species correlation network diagram. (f) LEfSe analysis. Data are presented as the mean ± SEM (n=3). *$P < 0.05$, **$P < 0.01$, ***$P < 0.001$. HCD_ND refers to the group of hypercholesterolemia model mice; v/i/uH6 refers to viable (vH6), heat-inactivated (iH6), and ultrasonically-lysed (uH6) bacteria cells; FMT1/2/3 refers to using feces from HCD_ND, Sim and vH6 groups, respectively.

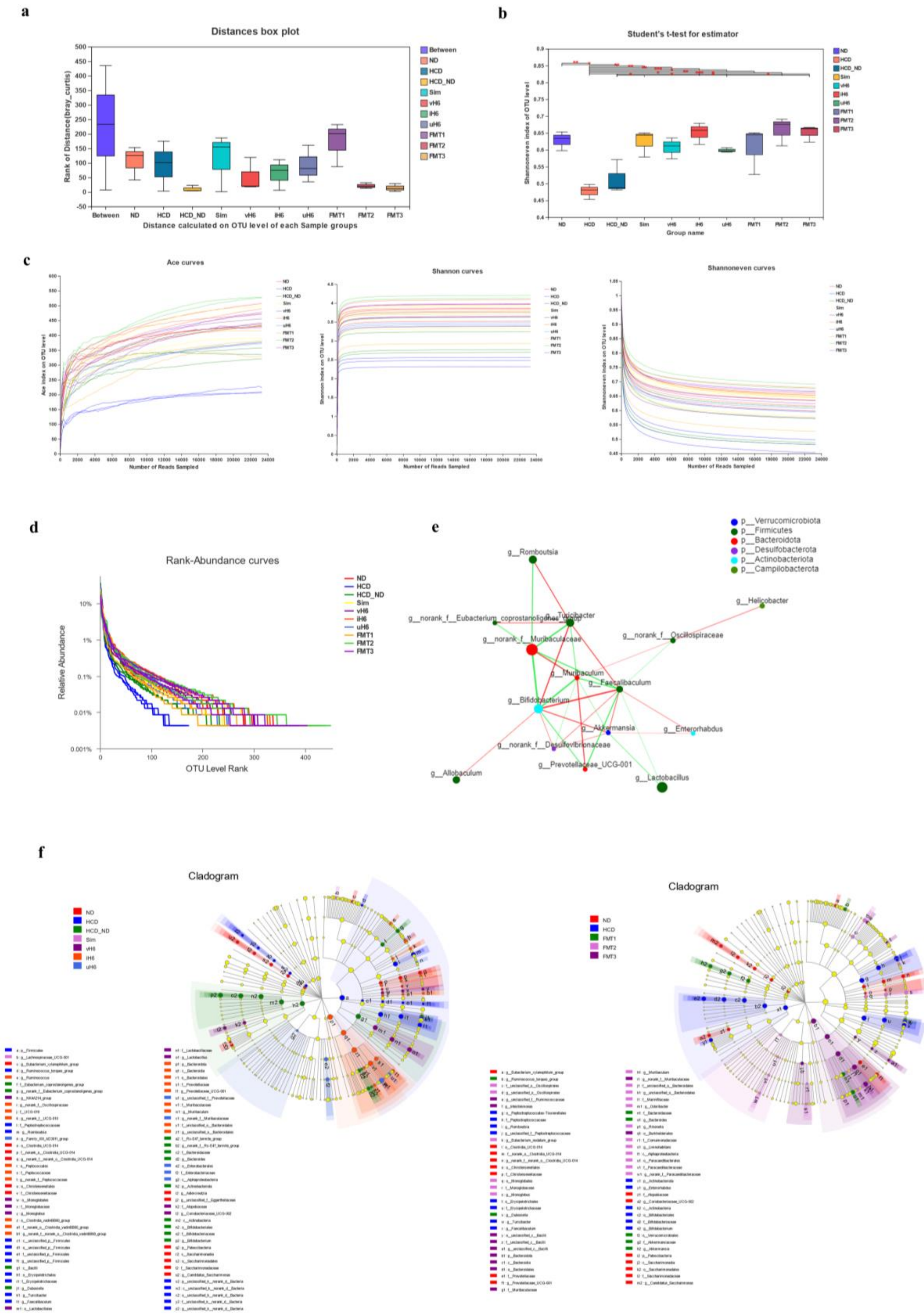
a
Distances box plot
Rank of Distance(bray_curtis)
Distance calculated on OTU level of each Sample groups
Between
ND
HCD
HCD_ND
Sim
vH6
iH6
uH6
FMT1
FMT2
FMT3
b
Student's t-test for estimator
Shannoneven index of OTU level
Group name
c
Ace curves
Shannon curves
Shannoneven curves
Number of Reads Sampled
d
Rank-Abundance curves
Relative Abundance
OTU Level Rank
e
p__Verrucomicrobiota
p__Firmicutes
p__Bacteroidota
p__Desulfobacterota
p__Actinobacteriota
p__Campilobacterota
g__Romboutsia
g__norank_f__Eubacterium_coprostanoligenes_group
g__Turicibacter
g__Helicobacter
g__norank_f__Muribaculaceae
g__norank_f__Oscillospiraceae
g__Muribaculum
g__Faecalibaculum
g__Bifidobacterium
g__Akkermansia
g__Enterorhabdus
g__norank_f__Desulfovibrionaceae
g__Prevotellaceae_UCG-001
g__Allobaculum
g__Lactobacillus
f
Cladogram
Cladogram

Supplementary figure 3. Effects of v/i/uH6 and FMT on intestinal metabolites in hypercholesterolemia mice. (a) Model validation of PLS-DA. (b) Heatmap of the profiling of all identified metabolites. (c-d) The relative content of vitamins-cofactors and amino acids detected by UPLC/Q-TOF-MS/MS based non-targeted metabolomics approach. (e) The content of acetic acid, propionic acid, butyric acid, isobutyric acid, valeric acid， isovaleric acid and total SCFAs. (f)KEGG pathway enrichment analysis of differential metabolites in HCD_ND vs. iH6， HCD_ND vs. uH6 groups and FMT1 vs. FMT3 groups. Data are presented as the mean ± SEM (n=3). $*P < 0.05$, $**P < 0.01$, $***P < 0.001$. HCD_ND refers to the group of hypercholesterolemia model mice; v/i/uH6 refers to viable (vH6), heat-inactivated (iH6), and ultrasonically-lysed (uH6) bacteria cells; FMT1/2/3 refers to using feces from HCD_ND, Sim and vH6 groups, respectively.

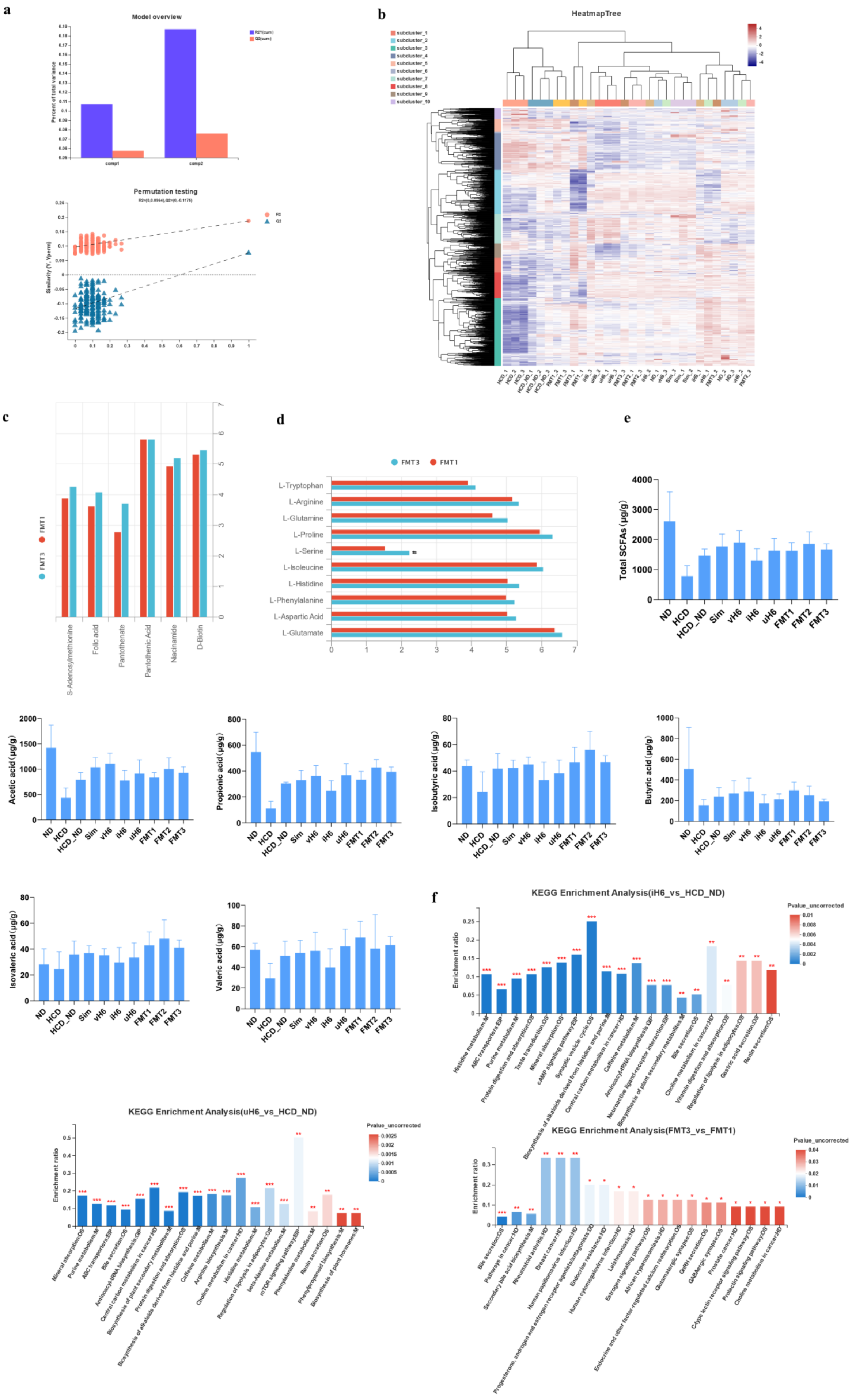
a
Model overview
Permutation testing
b
HeatmapTree
c
FMT1
FMT3
S-Adenosylmethionine
Folic acid
Pantothenate
Pantothenic Acid
Niacinamide
D-Biotin
d
FMT3
FMT1
L-Tryptophan
L-Arginine
L-Glutamine
L-Proline
L-Serine
L-Isoleucine
L-Histidine
L-Phenylalanine
L-Aspartic Acid
L-Glutamate
e
Total SCFAs (μg/g)
Acetic acid (μg/g)
Propionic acid (μg/g)
Isobutyric acid (μg/g)
Butyric acid (μg/g)
Isovaleric acid (μg/g)
Valeric acid (μg/g)
ND
HCD
HCD_ND
Sim
vH6
iH6
uH6
FMT1
FMT2
FMT3
f
KEGG Enrichment Analysis(iH6_vs_HCD_ND)
KEGG Enrichment Analysis(uH6_vs_HCD_ND)
KEGG Enrichment Analysis(FMT3_vs_FMT1)
Enrichment ratio
Pvalue_uncorrected

Supplementary figure 4. The correlation analysis of intestinal microbiota and microbial metabolites. (a-b) Redundancy analysis (RDA) of the correlation between intestinal microbiota and microbial metabolites (SCFAs and lipids) in mice given v/i/uH6. (c-g) RDA of the correlation between microbial community in mice treated with FMT3 and metabolites (vitamins-cofactors, amino acids, bile acids, SCFAs and lipids). Data are presented as the mean ± SEM (n=3). *$P < 0.05$, **$P < 0.01$, ***$P < 0.001$. HCD_ND refers to the group of hypercholesterolemia model mice; v/i/uH6 refers to viable (vH6), heat-inactivated (iH6), and ultrasonically-lysed (uH6) bacteria cells; FMT1/2/3 refers to using feces from HCD_ND, Sim and vH6 groups, respectively.

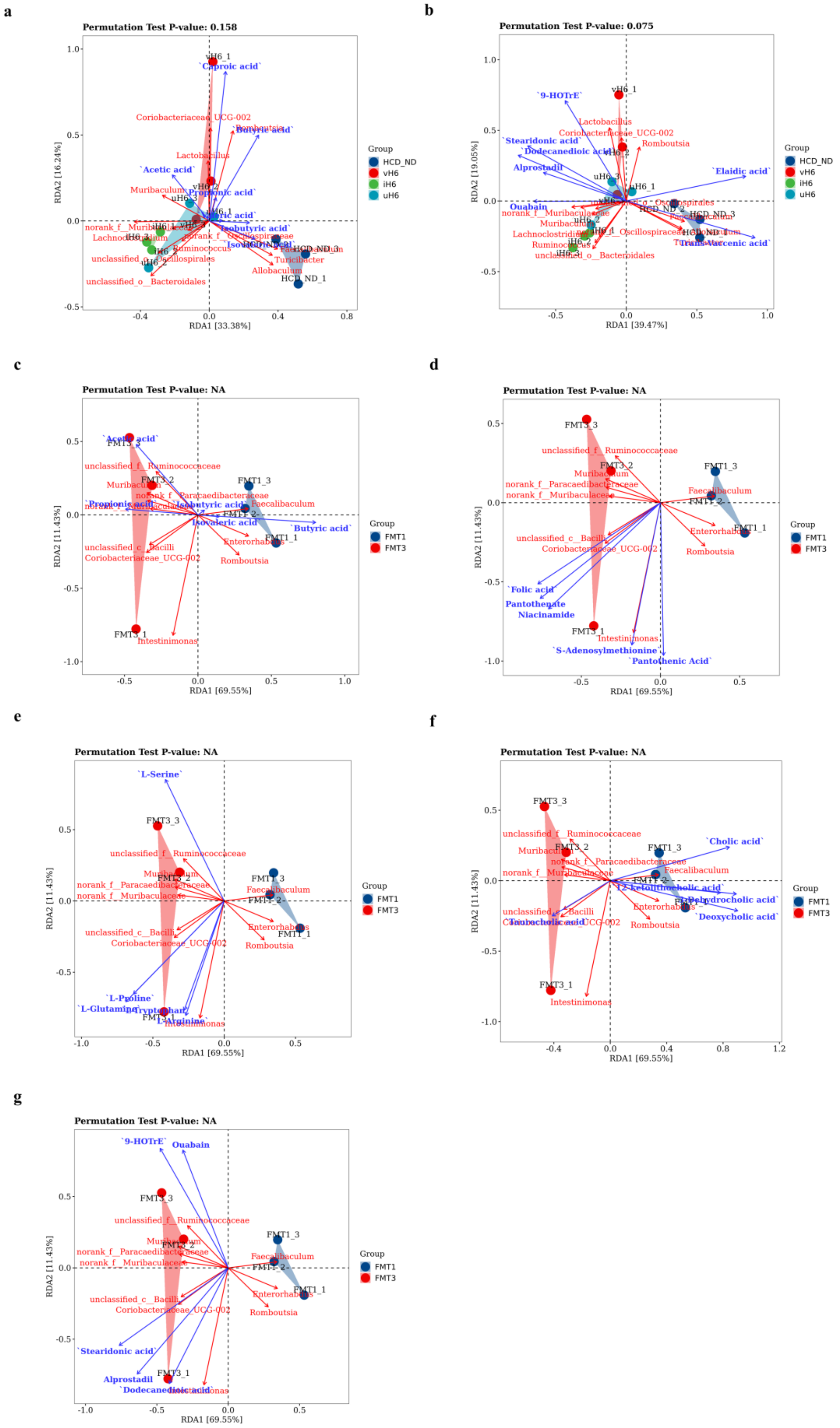
a
Permutation Test P-value: 0.158
RDA1 [33.38%]
RDA2 [16.24%]
`Caproic acid`
Coriobacteriaceae_UCG-002
`Butyric acid`
Lactobacillus
`Acetic acid`
`Propionic acid`
Muribaculum
`Isobutyric acid`
Lachnoclostridium
Ruminococcus
Turicibacter
Allobaculum
unclassified_o__Bacteroidales
Group
HCD_ND
vH6
iH6
uH6
b
Permutation Test P-value: 0.075
RDA1 [39.47%]
RDA2 [19.05%]
`9-HOTrE`
Lactobacillus
Coriobacteriaceae_UCG-002
Romboutsia
`Stearidonic acid`
`Dodecanedioic acid`
Alprostadil
`Elaidic acid`
Ouabain
norank_f__Muribaculaceae
Muribaculum
Lachnoclostridium
Ruminococcus
unclassified_o__Bacteroidales
`Trans-Vaccenic acid`
Group
HCD_ND
vH6
iH6
uH6
c
Permutation Test P-value: NA
RDA1 [69.55%]
RDA2 [11.43%]
`Acetic acid`
unclassified_f__Ruminococcaceae
Muribaculum
norank_f__Paracaedibacteraceae
`Propionic acid`
`Isobutyric acid`
Faecalibacterium
`Isovaleric acid`
`Butyric acid`
Enterorhabdus
unclassified_c__Bacilli
Coriobacteriaceae_UCG-002
Romboutsia
Intestinimonas
Group
FMT1
FMT3
d
Permutation Test P-value: NA
RDA1 [69.55%]
RDA2 [11.43%]
unclassified_f__Ruminococcaceae
Muribaculum
norank_f__Paracaedibacteraceae
norank_f__Muribaculaceae
Faecalibacterium
Enterorhabdus
unclassified_c__Bacilli
Coriobacteriaceae_UCG-002
Romboutsia
`Folic acid`
Pantothenate
Niacinamide
Intestinimonas
`S-Adenosylmethionine`
`Pantothenic Acid`
Group
FMT1
FMT3
e
Permutation Test P-value: NA
RDA1 [69.55%]
RDA2 [11.43%]
`L-Serine`
unclassified_f__Ruminococcaceae
Muribaculum
norank_f__Paracaedibacteraceae
norank_f__Muribaculaceae
Faecalibacterium
unclassified_c__Bacilli
Coriobacteriaceae_UCG-002
Enterorhabdus
Romboutsia
`L-Proline`
`L-Glutamine`
`L-Arginine`
Intestinimonas
Group
FMT1
FMT3
f
Permutation Test P-value: NA
RDA1 [69.55%]
RDA2 [11.43%]
unclassified_f__Ruminococcaceae
Muribaculum
`Cholic acid`
norank_f__Paracaedibacteraceae
norank_f__Muribaculaceae
Faecalibacterium
`12-ketolithocholic acid`
`Dehydrocholic acid`
Enterorhabdus
`Deoxycholic acid`
unclassified_c__Bacilli
`Taurocholic acid`
Romboutsia
Intestinimonas
Group
FMT1
FMT3
g
Permutation Test P-value: NA
RDA1 [69.55%]
RDA2 [11.43%]
`9-HOTrE`
Ouabain
unclassified_f__Ruminococcaceae
Muribaculum
norank_f__Paracaedibacteraceae
norank_f__Muribaculaceae
Faecalibacterium
Enterorhabdus
unclassified_c__Bacilli
Coriobacteriaceae_UCG-002
Romboutsia
`Stearidonic acid`
Alprostadil
`Dodecanedioic acid`
Intestinimonas
Group
FMT1
FMT3
